# Are Persons With Intellectual and Developmental Disabilities Being Embraced in Healthy Aging Interventions?

**DOI:** 10.1093/geroni/igab046.868

**Published:** 2021-12-17

**Authors:** Flavia Santos, Johanna Zurek, Matthew Janicki

**Affiliations:** 1 University College Dublin, Dublin, Dublin, Ireland; 2 Hochschule Doepfer, Cologne, Nordrhein-Westfalen, Germany; 3 University Of Illinois at Chicago, Chicago, Illinois, United States

## Abstract

We carried out a systematic review of healthy ageing interventions for adults with IDD. Twenty-three prospective studies including 2,398 men and women [average age: 44.3 years old] were found worldwide. Among them were only five RCTs. The designs usually were within or between subjects involving small sample sizes (ranging from 8 to 379 participants), mostly non-randomised or without follow up. We identified four thematic areas: Physical activity - nutrition and health (n = 10); Health education and health exams (n = 6); Social inclusion and community participation (n = 3); and Multi-components (n = 4). Overall, studies found effective outcomes, such as loss of body weight and improvement in nutritional habits, despite a few negative findings. We conclude that healthy ageing initiatives for people with IDD continue to be scarce, incipient, and sporadic. More research should embrace health promotion in people with IDD as a programme practice and public policy.

